# Outcomes of contralateral prophylactic mastectomy in relation to familial history: a decision analysis (BRCR-D-16-00033)

**DOI:** 10.1186/s13058-016-0752-y

**Published:** 2016-09-20

**Authors:** Kalatu R. Davies, Abenaa M. Brewster, Isabelle Bedrosian, Patricia A. Parker, Melissa A. Crosby, Susan K. Peterson, Yu Shen, Robert J. Volk, Scott B. Cantor

**Affiliations:** 1 Department of Health Services Research, The University of Texas MD Anderson Cancer Center, Unit 1444, P.O. Box 301402, Houston, TX 77230-1402 USA; 2 Department of Clinical Cancer Prevention, The University of Texas MD Anderson Cancer Center, Houston, TX USA; 3 Department of Breast Surgical Oncology, The University of Texas MD Anderson Cancer Center, Houston, TX USA; 4 Department of Psychiatry and Behavioral Sciences, Memorial Sloan-Kettering Cancer Center, New York, NY USA; 5 Department of Plastic Surgery, The University of Texas MD Anderson Cancer Center, Houston, TX USA; 6 Department of Behavioral Science, The University of Texas MD Anderson Cancer Center, Houston, TX USA; 7 Department of Biostatistics, The University of Texas MD Anderson Cancer Center, Houston, TX USA

**Keywords:** Contralateral breast cancer, Prophylactic mastectomy, Decision analysis, Breast cancer

## Abstract

**Background:**

Family history of breast cancer is associated with an increased risk of contralateral breast cancer (CBC) even in the absence of mutations in the breast cancer susceptibility genes *BRCA1/2.* We compared quality-adjusted survival after contralateral prophylactic mastectomy (CPM) with surveillance only (no CPM) among women with breast cancer incorporating the degree of family history.

**Methods:**

We created a microsimulation model for women with first-degree, second-degree, and no family history treated for a stage I, II, or III estrogen receptor (ER)-positive or ER-negative breast cancer at the ages of 40, 50, 60, and 70. The model incorporated a 10-year posttreatment period for risk of developing CBC and/or dying of the primary cancer or CBC. For each patient profile, we used 100,000 microsimulation trials to estimate quality-adjusted life expectancy for the clinical strategies CPM and no CPM.

**Results:**

CPM showed minimal improvement on quality-adjusted life expectancy among women age 50–60 with no or a unilateral first-degree or second-degree family history (decreasing from 0.31 to –0.06 quality-adjusted life-years (QALYs)) and was unfavorable for most subgroups of women age 70 with stage III breast cancer regardless of degree of family history (range –0.08 to –0.02 QALYs). Sensitivity analysis showed that the highest predicted benefit of CPM assuming 95 % risk reduction in CBC was 0.57 QALYs for a 40-year-old woman with stage I breast cancer who had a first-degree relative with bilateral breast cancer.

**Conclusions:**

Women age 40 with stage I breast cancer and a first-degree relative with bilateral breast cancer have a QALY benefit from CPM similar to that reported for *BRCA1/2* mutation carriers. For most subgroups of women, CPM has a minimal to no effect on quality-adjusted life expectancy, irrespective of family history of breast cancer.

## Background

Despite the minimal survival benefit of contralateral prophylactic mastectomy (CPM) and a declining incidence of contralateral breast cancer (CBC) [1] since 1998, the frequency of CPM has increased in the United States among women with sporadic, unilateral breast cancer [2, 3]. The reasons for the increased use of CPM include increased national rates of mastectomy compared to breast conserving surgery [2], the desire to reduce the risk of CBC, to improve survival, and to have peace of mind [4]. Although CPM may improve health outcomes for particular subgroups, specifically younger women with a *BRCA1/2* mutation, hereditary mutations account for only 5–10 % of cancers [5]. The additional surgery may thus be unnecessary for the majority of women diagnosed with breast cancer [6, 7].

Several decision analysis models have been developed for comparing CPM with surveillance only (no CPM) for the outcomes of life expectancy, quality-adjusted life expectancy, and cost-effectiveness [7–10]. For high-risk groups (i.e., women with a *BRCA1/2* mutation), CPM has been shown to be cost-effective compared with surveillance in terms of life expectancy [11]. In women without a *BRCA1/2* mutation, Portschy et al. [7] showed a less than 1 % 20-year survival benefit due to CPM for patients with stage I breast cancer, with an even smaller benefit for patients with stage II breast cancer.

Family history of breast cancer is considered to be an important risk factor for developing CBC even among women without mutations in the *BRCA1/2* breast cancer susceptibility gene [12, 13]. As the degree of family history of breast cancer increases, so does the risk of CBC. Noncarriers of a *BRCA1/2* mutation with any first-degree relative with bilateral breast cancer have CBC risk levels similar to those of *BRCA1/2* mutation carriers [14]. Several epidemiologic studies have shown that the frequency of CPM is higher among women with a family history of breast cancer [15, 16] and among women undergoing genetic testing even if they test negative for a mutation in *BRCA1/2* [17, 18]. However, the survival benefit of CPM in relation to family history of breast cancer, taking into consideration age, stage, and estrogen receptor (ER) status, has not been determined.

The aim of this study was to determine the impact of CPM on 20-year overall and disease-free survival and quality-adjusted life expectancy for women without a *BRCA1/2* mutation, taking into consideration age at diagnosis, disease stage, ER status, and degree of family history of breast cancer. We hypothesized that women with a higher degree of family history would experience the greatest long-term quality-adjusted survival benefit from CPM.

## Methods

### Model structure

**Fig. 1:**
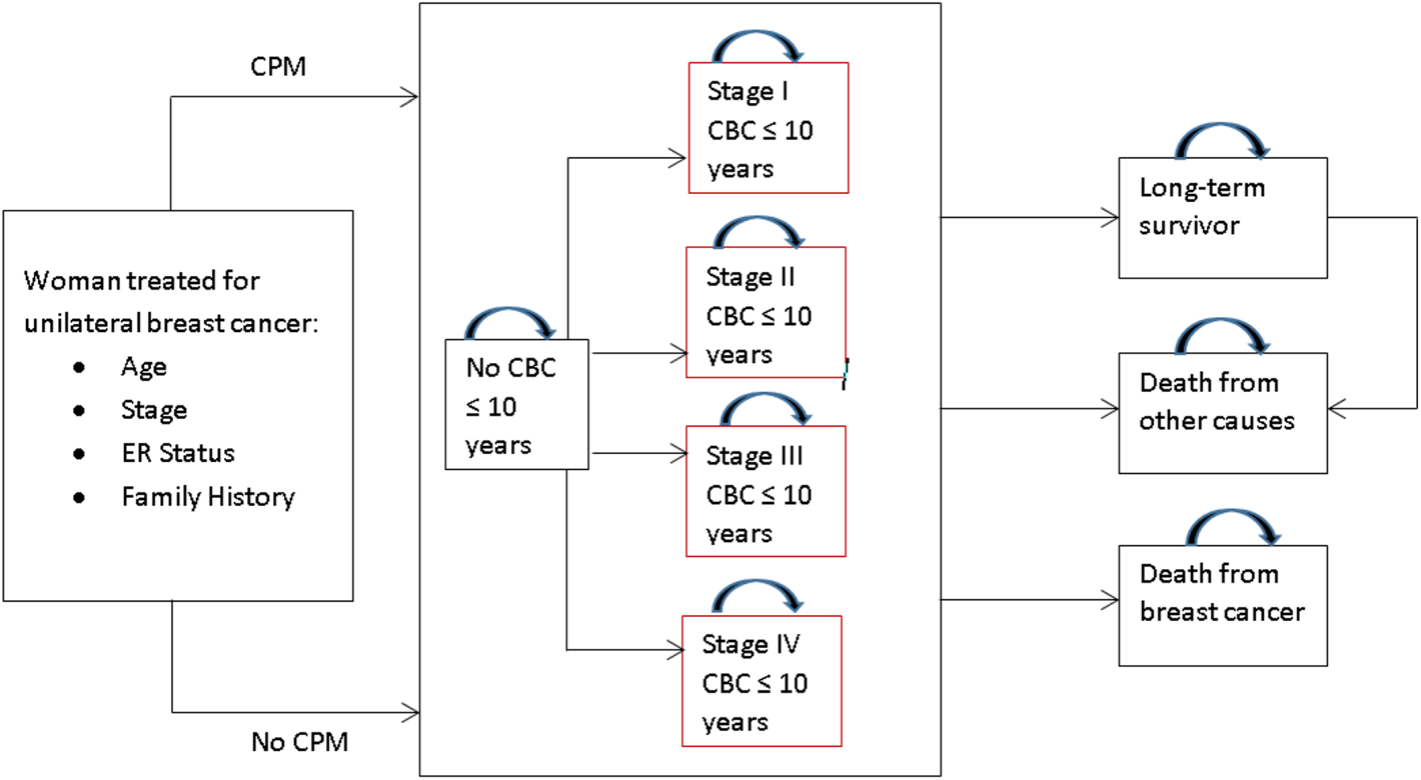
Markov model of outcomes of CPM and no CPM. Women in each Markov state (no CBC, CBC stages I, II, III, and IV) can transition to the “long-term survivor” health state after 10 years (cycles) without a breast cancer event or death and to the terminal states “death from other causes” and “death from breast cancer” during each cycle. CBC states outlined in red are health states with transition probabilities dependent on stage of CBC. *CPM* contralateral prophylactic mastectomy, *ER* estrogen receptor, *CBC* contralateral breast cancer (Color figure online)

We developed an individual-level state-transition model to simulate the long-term survival outcomes of women who undergo CPM and women who do not following a unilateral mastectomy or breast-conserving surgery of the primary breast cancer (Fig. 1). The model assumes a population of women with early-stage breast cancer without a hereditary breast cancer syndrome. Our analysis was conducted over a lifetime horizon, beginning at age 40, 50, 60, or 70 following treatment of the primary cancer. We assumed that patients received equivalent, standard treatment for the primary breast cancer in the CPM and non-CPM strategies. Since total complication rates for either unilateral or bilateral mastectomy with reconstruction are <2.5 % we did not consider them in the model [19]. Ten-year and 20-year overall and disease-free survival rates were determined by estimating life expectancy and disease-free life expectancy, respectively, over the corresponding time periods.

Each year, after treatment, the women may remain in a cancer-free (survivor) state, die of the primary cancer, develop a CBC, or die of other causes. The risk of CBC was incorporated into a 10-year period from the time of initial treatment. A 10-year period was chosen to look for the first event of a CBC because this time frame is sufficient to capture events in the natural progression of the disease [20]. If a CBC develops, the patient has an increased risk of dying from breast cancer, since the risks of dying from the primary and secondary cancers are considered additive [21]. Analogous to the 10-year risk period for development of a CBC or death from the primary cancer, patients are assumed to be at risk of death from the CBC for 10 years following its development and this annual risk is incorporated into the model with a 10-year post-CBC development risk period. Once the initial 10-year risk period for the index breast cancer ends, the overall mortality risk decreases because only the risk of dying from the CBC is considered.

We categorized patients who survive for 10 years without developing a CBC or since developing a CBC as long-term survivors. Long-term survival risks approach those of the general population after 10 years [20] and were considered the same for both the CPM group and the no-CPM group. An individual-level model was chosen for ease in tracking the length of time since treatment for the primary cancer or development of a CBC for precise estimation of the risk of death. We created the model using the decision analysis software TreeAge Pro 2014 (TreeAge Software, Inc., Williamstown, MA, USA).

### Probability estimates

Although many clinical characteristics, such as tumor grade or ER status, may influence the growth rate and the metastatic potential of small tumors [22], the stage of a primary tumor at diagnosis is a key indicator of prognosis, particularly for early-stage cancers. Thus, our model incorporates annual breast cancer-specific mortality rates extrapolated from 10-year disease-specific risks of death for patients with stage I–III cancers that were derived from the relative survival curves in the Surveillance, Epidemiology, and End Results database as presented in Portschy et al. [7]. Age-specific mortality rates were obtained from US life tables [23].

The annual risk of developing a CBC is thought to be 0.5–0.75 % [6, 24–29]. However, this may be an overestimate due to the now widespread use of adjuvant systemic therapy. Adjuvant hormonal therapies for women with ER-positive tumors, including tamoxifen for premenopausal women and aromatase inhibitors for postmenopausal women, have been shown to reduce CBC risk by as much as 50 % [2, 3]. Thus, the risk of CBC is lower for patients with ER-positive than ER-negative breast cancers. In a meta-analysis performed by the Early Breast Cancer Trialists’ Collaborative Group, the 15-year incidence of CBC was 6.5 % in women with ER-positive disease who were randomized to tamoxifen therapy and was approximately 7.1 % in women with ER-negative disease regardless of use of tamoxifen [30]. These rates were converted to annual probabilities, assuming the incidence rates were constant over the 15-year interval, resulting in 0.4 % and 0.5 % annual risks of developing CBC for ER-positive and ER-negative patients, respectively. We used these estimates to determine the ratio of the risk of developing CBC in ER-negative patients to the risk in ER-positive patients.

The risks of CBC according to family history are an average annual risk of developing CBC estimated from a population-based case–control study of women without *BRCA* mutation who had a primary breast cancer in four US cancer registries. The study population may have included women who were treated with a unilateral mastectomy or breast-conserving surgery followed by radiation and/or chemotherapy [13].

To incorporate both ER status and family history into the risk for developing CBC, we assumed that the average risk for each degree of family history (none, second-degree relative, first-degree relative with unilateral breast cancer, and first-degree relative with bilateral breast cancer) is a weighted average of risks for patients with and ER-negative tumors. Then, using the proportions of ER-positive and ER-negative tumors, estimated to be 75 % and 25 %, respectively [12], and the ratio of average CBC risks according to ER status (Table 1), we estimated the risk for developing CBC for patients with ER-negative tumors and patients with ER-positive tumors and at varying degrees of family history.

**Table 1:** Base Case Probabilities, Utilities, and Ranges Used in Sensitivity Analysis

Variable	Base Case	Range	Reference
Annual risk of CBC			[13]
No family history of breast cancer	0.0047	0.0041–0.0052	
Second-degree relative only	0.0061	0.0470–0.0900	
First-degree relative, unilateral	0.0090	0.0063–0.0121	
First-degree relative, bilateral	0.0168	0.0088–0.0330	
BRCA 1/2 mutation carriers	0.0184	0.0160–0.0213	
ER-negative/ER-positive risk ratio	1.25	1.10–2.00	[6, 7, 12, 13, 24–30]
CBC risk reduction after CPM	0.95	0.75–1.00	[2, 21, 32, 33]
Risk of developing CBC stage I-IV			[21]
Stage I	0.66		
Stage II	0.25		
Stage III	0.05		
Stage IV	0.04		
10-year disease-specific risk of dying			[7]
Stage I	0.018		
Stage II	0.231		
Stage III	0.592		
Stage IV	0.911		
Risk of dying of other causes	Age specific		[23]
			[8, 31]
First year after treatment			
CPM	0.70	0.41–0.95	
No CPM	0.90	0.87–1.00	
No CBC			
Years 2-5	0.79	0.45–1.00	
Year 6 and beyond	0.84	0.77–1.00	
CBC			
New CBC	0.73	0.58–1.00	
Years 2-5	0.79	0.45–1.00	
Year 6 and beyond	0.84	0.77–1.00	
Long-term survivor	0.84	0.77–1.00	

*CPM* contralateral prophylactic mastectomy, *CBC* contralateral breast cancer

The stage distribution of a CBC was derived from a study of the Oregon State Cancer Registry database by Quan et al. [21]. In that database, 9 years of patient cases of breast cancer were queried and over 90 % of CBCs were determined to be early stage. We estimated that the probabilities of developing stage I, II, III, and IV CBC were 66 %, 25 %, 5 %, and 4 %, respectively. The risk of death associated with a CBC was estimated to be the same as the risk of death associated with a primary cancer of the same stage and was added to the risk associated with the primary breast cancer. Patients were considered at risk for developing CBC within 10 years after treatment of the primary breast cancer.

### Utility estimates

We incorporated quality of life for each health state using estimates of utilities reported in the literature [8, 31]. Utilities typically measure quality of life on a 0–1 scale anchored by death and perfect health. Reduced quality of life is associated with treatment and disease health states [31]. The impact of each treatment strategy was thus measured in quality-adjusted life years (QALYs), which are years lived equivalent to perfect health. We identified two primary health states— unilateral mastectomy or breast-conserving surgery (no CPM), and bilateral mastectomy involving a CPM—for which utility adjustments needed to be made. We assumed the initial utility of unilateral mastectomy or breast-conserving surgery to be 0.90 and the initial utility of bilateral mastectomy to be 0.70, because of the greater initial postsurgery disutility associated with bilateral mastectomy. If no CBC developed during the first year, in years 2–5 after surgery the utilities were assumed to be 0.79 for both health states; beyond year 5, the utility for both health states was assumed to be 0.84. If a CBC developed at any time during the 10-year period, the utility value was changed to 0.73 for 2 years following the CBC diagnosis. Between years 2 and 5, the utility value for the CBC health state was increased to 0.79. As with the primary (no-CBC) health state, this value was increased to 0.84 beyond year 5.

### Sensitivity analysis

We conducted a sensitivity analysis of base case parameter estimates of reductions in the risk of developing CBC after CPM. The variable means and ranges were established on the basis of published estimates (Table 1). For our base-case analysis (Tables 2, 3, 4, 5), we estimated that CPM reduces the risk of developing CBC by approximately 95 % [2, 21, 32, 33]. In our sensitivity analysis, we used a CBC risk reduction in the range of 75–100 % for each degree of family history. To perform a sensitivity analysis on the utility values, we conducted the sensitivity analysis of CBC risk and CPM-associated risk reduction with the utility associated with each health state set to 1 (i.e., no health decrements in the model), resulting in life expectancy as the outcome. In addition, we conducted a threshold analysis of the utility associated with CPM during the year immediately following the surgery. In the probabilistic sensitivity analysis, to evaluate uncertainty in the model parameters, the mean and range were used to calculate the parameter distribution values. We assumed a beta distribution for all probabilities, a table distribution for tumor stage based on the frequency, and a gamma distribution for the risk ratio of ER-negative to ER-positive, and performed 10,000 Monte Carlo simulations.

**Table 2:** Predicted Differences in Life Expectancy by Degree of Family History of Breast Cancer

Patient and Disease Characteristics			Life Years											
			No Family History			Breast Cancer in a Second-Degree Relative			Breast Cancer in a First-Degree Relative					
									Unilateral			Bilateral		
Age (years)	ER Status	Stage	No CPM	CPM	Δ	No CPM	CPM	Δ	No CPM	CPM	Δ	No CPM	CPM	Δ
40	Positive	I	41.43	41.63	0.21	41.37	41.63	0.26	41.25	41.63	0.38	40.93	41.61	0.68
40	Negative	I	41.28	41.51	0.24	41.21	41.51	0.30	41.06	41.50	0.45	40.66	41.48	0.82
40	Positive	II	32.85	32.99	0.14	32.80	32.98	0.18	32.69	32.98	0.28	32.43	32.97	0.54
40	Negative	II	32.74	32.94	0.20	32.68	32.94	0.25	32.58	32.93	0.35	32.27	32.91	0.65
40	Positive	III	18.55	18.63	0.08	18.53	18.63	0.10	18.49	18.63	0.14	18.37	18.62	0.25
40	Negative	III	18.45	18.57	0.11	18.42	18.56	0.14	18.36	18.56	0.20	18.20	18.54	0.34
50	Positive	I	32.27	32.43	0.15	32.22	32.42	0.20	32.13	32.42	0.29	31.90	32.41	0.51
50	Negative	I	32.30	32.50	0.20	32.24	32.50	0.26	32.12	32.49	0.37	31.85	32.47	0.62
50	Positive	II	25.97	26.10	0.12	25.94	26.09	0.16	25.87	26.09	0.23	25.69	26.08	0.39
50	Negative	II	25.93	26.08	0.16	25.88	26.08	0.20	25.82	26.08	0.26	25.60	26.06	0.46
50	Positive	III	15.13	15.18	0.05	15.11	15.18	0.07	15.08	15.18	0.10	14.99	15.18	0.19
50	Negative	III	15.08	15.15	0.06	15.07	15.15	0.08	15.03	15.14	0.12	14.91	15.14	0.23
60	Positive	I	23.84	23.93	0.09	23.82	23.93	0.11	23.76	23.93	0.17	23.61	23.92	0.31
60	Negative	I	23.88	23.99	0.11	23.84	23.99	0.15	23.76	23.98	0.22	23.59	23.97	0.38
60	Positive	II	19.39	19.47	0.08	19.36	19.47	0.11	19.32	19.47	0.14	19.19	19.46	0.27
60	Negative	II	19.46	19.53	0.08	19.43	19.53	0.10	19.39	19.53	0.14	19.25	19.52	0.26
60	Positive	III	11.92	11.96	0.03	11.91	11.96	0.04	11.89	11.96	0.07	11.83	11.95	0.13
60	Negative	III	11.84	11.89	0.05	11.82	11.89	0.07	11.78	11.89	0.11	11.72	11.88	0.17
70	Positive	I	16.14	16.19	0.05	16.13	16.19	0.06	16.09	16.19	0.09	16.02	16.18	0.17
70	Negative	I	16.09	16.15	0.06	16.07	16.15	0.08	16.04	16.15	0.11	15.95	16.14	0.19
70	Positive	II	13.50	13.53	0.04	13.49	13.53	0.05	13.45	13.54	0.08	13.39	13.53	0.14
70	Negative	II	13.50	13.55	0.05	13.49	13.55	0.06	13.45	13.54	0.10	13.36	13.54	0.18
70	Positive	III	8.78	8.79	0.02	8.77	8.79	0.02	8.75	8.79	0.04	8.72	8.79	0.07
70	Negative	III	8.78	8.80	0.03	8.77	8.80	0.04	8.76	8.80	0.05	8.73	8.80	0.07

*ER* estrogen receptor, *CPM* contralateral prophylactic mastectomy, Δ, difference (CPM-No CPM); CBC risk reduction = 95% (base-case)

**Table 3:** Predicted Differences in Quality-Adjusted Life Expectancy by Degree of Family History of Breast Cancer

Patient and Disease Characteristics			Quality-Adjusted Life-Years											
			No Family History			Breast Cancer in a Second-Degree Relative			Breast Cancer in a First-Degree Relative					
									Unilateral			Bilateral		
Age (years)	ER Status	Stage	No CPM	CPM	Δ	No CPM	CPM	Δ	No CPM	CPM	Δ	No CPM	CPM	Δ
40	Positive	I	34.61	34.70	0.09	34.56	34.70	0.14	34.45	34.70	0.25	34.16	34.68	0.52
40	Negative	I	34.48	34.60	0.12	34.43	34.60	0.17	34.28	34.59	0.31	33.92	34.57	0.65
40	Positive	II	27.42	27.45	0.03	27.38	27.45	0.07	27.28	27.44	0.16	27.04	27.43	0.40
40	Negative	II	27.33	27.41	0.08	27.28	27.41	0.13	27.18	27.40	0.23	26.89	27.39	0.50
40	Positive	III	15.45	15.42	-0.03	15.42	15.42	0.00	15.38	15.41	0.03	15.28	15.41	0.14
40	Negative	III	15.36	15.36	0.01	15.33	15.36	0.03	15.27	15.36	0.09	15.12	15.34	0.22
50	Positive	I	26.92	26.97	0.05	26.88	26.97	0.09	26.79	26.96	0.17	26.58	26.95	0.38
50	Negative	I	26.94	27.03	0.09	26.89	27.03	0.14	26.78	27.02	0.25	26.52	27.01	0.49
50	Positive	II	21.65	21.66	0.02	21.62	21.66	0.05	21.55	21.66	0.11	21.38	21.65	0.27
50	Negative	II	21.61	21.65	0.05	21.57	21.65	0.09	21.50	21.65	0.15	21.29	21.63	0.34
50	Positive	III	12.57	12.52	-0.05	12.55	12.52	-0.03	12.52	12.52	0.00	12.43	12.52	0.08
50	Negative	III	12.53	12.49	-0.04	12.51	12.49	-0.02	12.47	12.49	0.02	12.36	12.49	0.13
60	Positive	I	19.85	19.84	-0.01	19.82	19.84	0.01	19.76	19.83	0.07	19.61	19.83	0.21
60	Negative	I	19.87	19.88	0.01	19.83	19.88	0.05	19.76	19.88	0.12	19.58	19.87	0.28
60	Positive	II	16.12	16.10	-0.02	16.10	16.10	0.00	16.06	16.10	0.04	15.93	16.09	0.17
60	Negative	II	16.17	16.15	-0.02	16.15	16.15	0.00	16.10	16.15	0.05	15.97	16.14	0.17
60	Positive	III	9.88	9.82	-0.06	9.87	9.81	-0.05	9.84	9.81	-0.03	9.78	9.81	0.03
60	Negative	III	9.80	9.76	-0.04	9.79	9.76	-0.03	9.75	9.76	0.01	9.68	9.75	0.07
70	Positive	I	13.38	13.34	-0.05	13.37	13.34	-0.03	13.33	13.33	0.00	13.25	13.33	0.08
70	Negative	I	13.34	13.30	-0.03	13.32	13.30	-0.02	13.28	13.30	0.02	13.18	13.29	0.11
70	Positive	II	11.18	11.12	-0.06	11.16	11.12	-0.05	11.13	11.12	-0.01	11.06	11.12	0.05
70	Negative	II	11.18	11.13	-0.05	11.16	11.13	-0.03	11.12	11.13	0.01	11.03	11.12	0.09
70	Positive	III	7.24	7.16	-0.08	7.24	7.16	-0.07	7.22	7.16	-0.06	7.18	7.16	-0.02
70	Negative	III	7.24	7.17	-0.07	7.23	7.17	-0.06	7.22	7.17	-0.05	7.18	7.17	-0.01

*ER* estrogen receptor, *CPM* contralateral prophylactic mastectomy, Δ, difference (CPM-No CPM); CBC risk reduction = 95% (base-case)

**Table 4:** Expected Incidence of Contralateral Breast Cancer (CBC) by Degree of Family History of Breast Cancer

Patient and Disease Characteristics			No. of CBCs/1000 women over 10 years											
			No Family History			Breast Cancer in a Second-Degree Relative			Breast Cancer in a First-Degree Relative					
									Unilateral			Bilateral		
Age (years)	ER Status	Stage	No CPM	CPM	Δ	No CPM	CPM	Δ	No CPM	CPM	Δ	No CPM	CPM	Δ
40	Positive	I	46	2	44	59	3	56	86	4	82	158	9	149
40	Negative	I	58	3	55	74	4	70	108	6	102	193	10	183
40	Positive	II	41	2	39	52	3	49	77	4	73	137	8	129
40	Negative	II	49	2	47	64	3	61	93	5	88	169	9	160
40	Positive	III	29	2	27	36	2	34	54	3	51	98	5	93
40	Negative	III	36	2	34	46	2	44	67	3	64	121	7	114
50	Positive	I	46	2	44	59	3	56	86	5	81	154	8	146
50	Negative	I	57	3	54	73	4	69	107	6	101	191	11	180
50	Positive	II	40	2	38	51	2	49	75	3	72	134	7	127
50	Negative	II	49	2	47	63	3	60	92	5	87	166	9	157
50	Positive	III	28	1	27	36	2	34	53	3	50	95	5	90
50	Negative	III	35	2	33	45	2	43	66	3	63	119	6	113
60	Positive	I	45	2	43	57	3	54	83	4	79	152	8	144
60	Negative	I	54	3	51	70	4	66	102	5	97	183	10	173
60	Positive	II	38	2	36	49	2	47	72	4	68	132	7	125
60	Negative	II	49	3	46	62	3	59	90	5	85	163	9	154
60	Positive	III	28	1	27	36	2	34	53	3	50	95	5	90
60	Negative	III	35	2	33	45	2	43	66	3	63	117	6	111
70	Positive	I	41	2	39	53	3	50	77	4	73	141	8	133
70	Negative	I	52	3	49	66	3	63	96	5	91	173	9	164
70	Positive	II	36	2	34	47	3	44	68	4	64	121	7	114
70	Negative	II	45	2	43	57	3	54	83	4	79	150	8	142
70	Positive	III	26	1	25	34	1	33	50	2	48	89	4	85
70	Negative	III	32	2	30	41	2	39	59	3	56	108	6	102

*ER* estrogen receptor*, CPM* contralateral prophylactic mastectomy, Δ, difference (No CPM-CPM); CBC risk reduction = 95% (base-case)

**Table 5:** 10 and 20-year Overall and Disease-Free Survival Rate Differences by Degree of Family History of Breast Cancer

Patient and Disease Characteristics			10 and 20-year absolute overall survival (OS) and disease-free survival (DFS) rate difference (%)															
			No Family History				Breast Cancer IN A Second-Degree Relative				Breast Cancer in a First-Degree Relative							
											Unilateral				Bilateral			
Age (years)	ER Status	Stage	10, 20yr OS Δ%		10, 20yr DFS Δ%		10, 20yr OS Δ%		10, 20yr DFS Δ%		10, 20yr OS Δ%		10, 20yr DFS Δ%		10, 20yr OS Δ%		10, 20yr DFS Δ%	
40	Positive	I	0.09	0.29	1.79	2.97	0.12	0.36	2.31	3.81	0.18	0.54	3.42	5.61	0.32	0.96	6.28	10.24
40	Negative	I	0.12	0.35	2.22	3.73	0.14	0.43	2.83	4.76	0.21	0.65	4.18	6.97	0.43	1.21	7.74	12.59
40	Positive	II	0.07	0.20	1.55	2.39	0.09	0.27	2.01	3.07	0.15	0.43	2.98	4.56	0.29	0.79	5.39	8.16
40	Negative	II	0.09	0.27	1.91	2.92	0.12	0.36	2.47	3.77	0.17	0.50	3.65	5.54	0.35	0.94	6.75	10.09
40	Positive	III	0.04	0.11	1.03	1.31	0.06	0.15	1.32	1.68	0.10	0.21	1.95	2.47	0.16	0.38	3.54	4.47
40	Negative	III	0.07	0.17	1.32	1.69	0.09	0.20	1.70	2.15	0.12	0.29	2.43	3.08	0.21	0.52	4.42	5.54
50	Positive	I	0.10	0.29	1.81	2.91	0.13	0.38	2.31	3.72	0.18	0.54	3.37	5.43	0.32	0.94	6.18	9.77
50	Negative	I	0.14	0.36	2.22	3.59	0.17	0.46	2.83	4.59	0.26	0.69	4.20	6.75	0.45	1.18	7.69	12.17
50	Positive	II	0.09	0.24	1.52	2.30	0.12	0.31	1.97	2.97	0.18	0.44	2.92	4.37	0.31	0.79	5.28	7.81
50	Negative	II	0.13	0.30	1.92	2.88	0.15	0.38	2.47	3.70	0.20	0.52	3.56	5.31	0.36	0.92	6.45	9.57
50	Positive	III	0.04	0.10	0.97	1.24	0.05	0.13	1.25	1.61	0.08	0.19	1.84	2.31	0.16	0.36	3.38	4.23
50	Negative	III	0.07	0.13	1.25	1.56	0.08	0.17	1.62	2.02	0.12	0.25	2.40	2.97	0.22	0.47	4.34	5.34
60	Positive	I	0.09	0.25	1.72	2.65	0.11	0.31	2.20	3.38	0.17	0.46	3.23	4.92	0.33	0.84	6.00	9.00
60	Negative	I	0.11	0.30	2.08	3.16	0.15	0.39	2.75	4.13	0.22	0.58	4.03	6.06	0.40	1.04	7.38	10.97
60	Positive	II	0.08	0.22	1.47	2.07	0.10	0.28	1.88	2.66	0.15	0.38	2.78	3.90	0.30	0.72	5.15	7.19
60	Negative	II	0.08	0.20	1.88	2.64	0.11	0.26	2.37	3.33	0.14	0.36	3.44	4.82	0.28	0.69	6.32	8.75
60	Positive	III	0.04	0.10	0.98	1.17	0.06	0.13	1.27	1.53	0.08	0.20	1.85	2.21	0.16	0.35	3.39	3.99
60	Negative	III	0.07	0.15	1.26	1.51	0.09	0.18	1.62	1.94	0.15	0.31	2.39	2.86	0.24	0.49	4.28	5.04
					Ygy6													
70	Positive	I	0.09	0.19	1.61	2.06	0.11	0.24	2.04	2.64	0.16	0.35	2.98	3.84	0.28	0.62	5.54	7.03
70	Negative	I	0.12	0.23	2.01	2.58	0.14	0.29	2.57	3.29	0.21	0.42	3.74	4.78	0.37	0.75	6.83	8.64
70	Positive	II	0.07	0.14	1.32	1.59	0.09	0.18	1.71	2.08	0.15	0.31	2.51	3.06	0.27	0.52	4.60	5.54
70	Negative	II	0.08	0.18	1.65	2.03	0.10	0.23	2.12	2.58	0.17	0.37	3.11	3.79	0.31	0.67	5.70	6.85
70	Positive	III	0.05	0.07	0.91	0.93	0.07	0.10	1.21	1.23	0.11	0.16	1.76	1.78	0.18	0.29	3.21	3.22
70	Negative	III	0.05	0.10	1.08	1.12	0.06	0.13	1.40	1.46	0.09	0.17	2.06	2.12	0.18	0.30	3.80	3.86

*ER* estrogen receptor, Δ%, difference %; CBC risk reduction = 95% (base-case)

## Results

### Effect of CPM on life expectancy by degree of family history of breast cancer

CPM was associated with gains (0.02–0.82 years) in life expectancy for all patient subgroups (Table 2)*.* Greater benefits were seen in patients age < 60, ER-negative status, disease stage I or II, and having a first-degree family history of breast cancer, especially with bilateral breast cancer. The benefits of CPM decreased in patients with characteristics associated with a greater risk of dying of primary breast cancer or other causes: higher disease stage and older age.

**Fig. 2:**
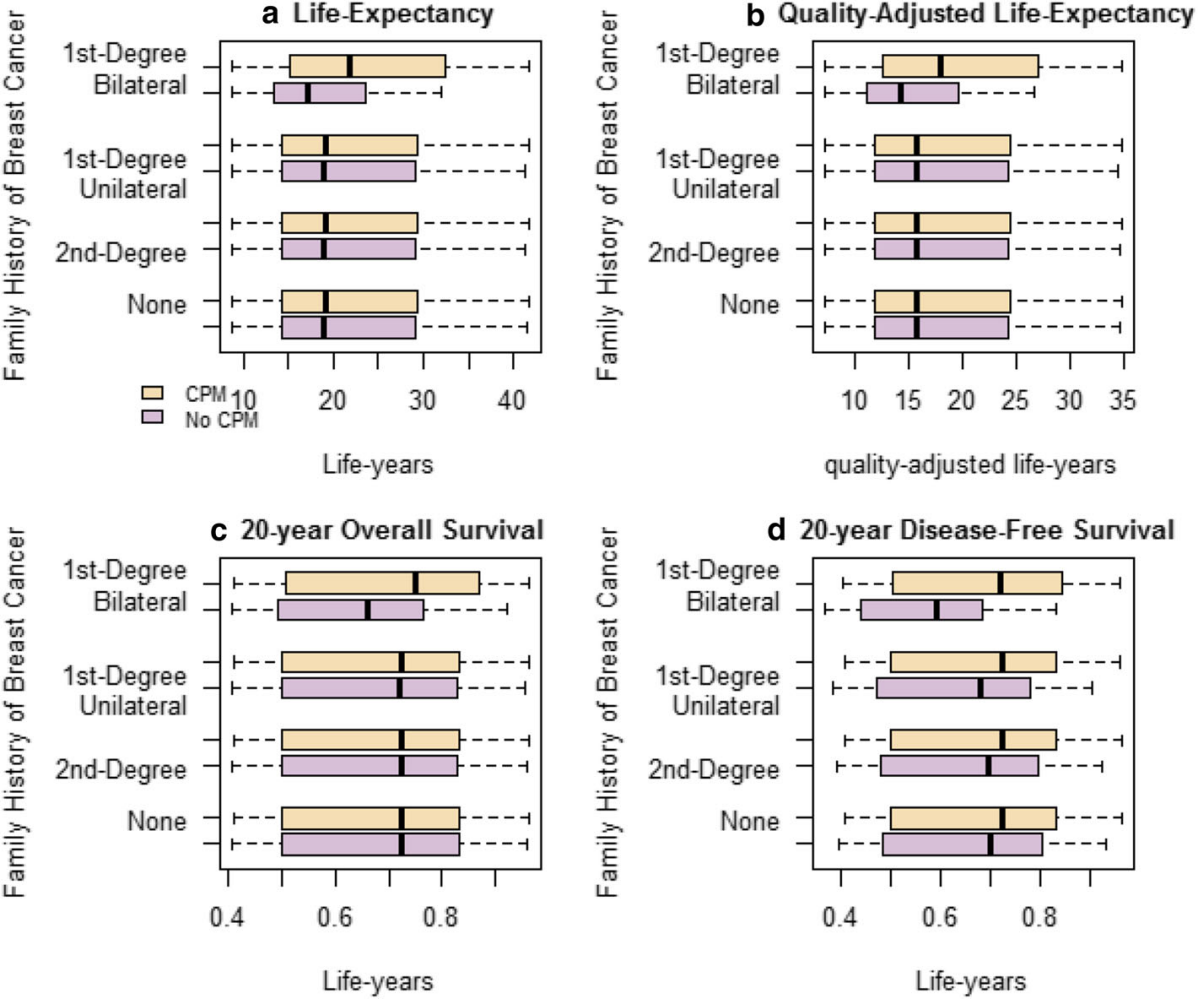
Boxplot comparisons of survival outcomes. **a** Life expectancy, **b** quality-adjusted life expectancy, **c** 20-year overall survival, and **d** 20-year disease-free survival with and without contralateral prophylactic mastectomy (*CPM*) in relation to family history of breast cancer

**Fig. 3:**
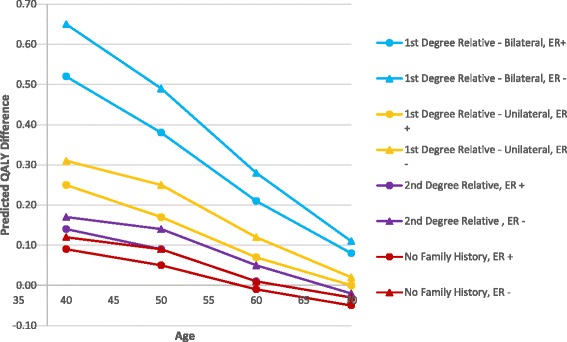
Predicted differences in quality-adjusted survival benefit by age, ER status, and family history of breast cancer. Quality-adjusted survival benefit of CPM in women with stage I breast cancer in relation to age, ER status, and family history. *ER* estrogen receptor, *QALY* quality-adjusted life year

**Fig. 4:**
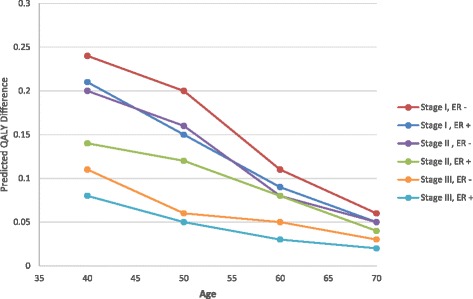
Predicted differences in quality-adjusted survival benefit by age, ER status, and primary breast cancer stage in women with no family history of breast cancer. Quality-adjusted survival benefit of CPM in women with no family history of breast cancer in relation to age, ER status, and primary breast cancer stage. *ER* estrogen receptor, *QALY* quality-adjusted life year

After quality-of-life adjustments were incorporated, women age 40–50 with a first-degree relative with breast cancer tended to have a positive benefit with CPM (Fig. 2). However, the benefit was minimal to negative for those with either no or a second-degree family history or stage III disease. CPM had an overwhelmingly minimal to negative benefit for women age 60–70 with stage I–III disease and no family history of breast cancer. Women age 70 with stage III disease had a negative benefit from CPM regardless of the degree of family history of breast cancer. For the 40–70 age range, the benefit of CPM compared with no CPM decreased from 0.12 to –0.08 to QALYs for women with no family history of breast cancer, from 0.17 to –0.07 QALYs for women with only a second-degree relative with breast cancer, from 0.31 to –0.06 QALYs for women with a first-degree relative with unilateral breast cancer, and from 0.65 to –0.02 QALYs for women with a first-degree relative with bilateral breast cancer (Table 3). For all age groups, women with ER-negative breast cancers had a greater QALY benefit from CPM (or less negative benefit for women age 60–70) than patients with ER-positive breast cancers, irrespective of family history (Fig. 3). A similar relationship was also observed for patients with ER-negative versus ER-positive breast cancer irrespective of stage of the primary cancer (Fig. 4).

### Effect of CPM on CBC, overall survival, and disease-free survival by degree of family history of breast cancer

A greater number of CBCs occurred in the group who did not have CPM. With CPM, the overall range of the rate of CBCs was reduced from 26–193 per 1000 women to 1–11 per 1000 women (Table 4).

Generally, the prevention of CBC had greater survival benefit for younger women. However, for women age 50 with stage I or II primary breast cancer there was the same or slightly greater 10-year and 20-year survival benefit associated with CPM compared with those age 40, irrespective of family history (Table 5). There may thus be an additive effect between the breast cancer mortality risk and increased background mortality risk at age 50 compared with age 40. With the older age groups, however, background mortality outweighed any CBC mortality risk.

For all age groups, the greatest reduction in the range of the rates of CBC as a result of CPM occurred in women with a higher degree of family history and with ER-negative tumors (Table 4). The maximum 20-year absolute overall survival rate benefit for CPM versus no CPM was 0.36 % for women with no family history of breast cancer, 0.46 % for women with only a second-degree relative with breast cancer, 0.69 % for women with a first-degree relative with unilateral breast cancer, and 1.21 % for women with a first-degree relative with bilateral breast cancer (Table 5, Fig. 2c). The maximum 20-year absolute disease-free survival rate benefit for CPM versus no CPM was 3.73 % for women with no family history of breast cancer, 4.76 % for women with only a second-degree relative with breast cancer, 6.97 % for women with a first-degree relative with unilateral breast cancer, and 12.59 % for women with a first-degree relative with bilateral breast cancer (Table 5, Fig. 2d).

### Sensitivity analysis of differences in quality-adjusted life expectancy by degree of family history of breast cancer

**Fig. 5:**
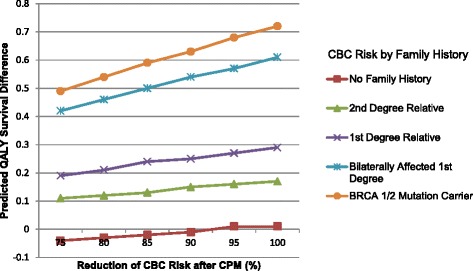
Sensitivity analysis of predicted differences in quality-adjusted survival. Quality-adjusted survival in 40-year-old women with stage I breast cancer and various annual probabilities of developing contralateral breast cancer (*CBC*), in relation to family history and effectiveness of contralateral prophylactic mastectomy (*CPM*). *QALY* quality-adjusted life year

In our model, the risk of developing CBC varied according to family history. The predicted absolute differences in quality-adjusted life expectancy given the CBC risk associated with the degree of family history and *BRCA1/2* mutation status are shown in Fig. 5. Greater benefits of CPM were predicted for women with greater risk of developing CBC. With the CBC risk reduction assumed to be 100 %, the maximum absolute benefit for a 40-year-old woman with stage I breast cancer was 0.61 QALYs, and the annual probability of developing a CBC for the aforementioned woman with a first-degree relative with bilateral breast cancer was 1.68 %. In comparison, the maximum absolute benefit for a 40-year-old woman with stage I breast cancer and *BRCA1/2* mutation was 0.72 QALYs, and the annual probability of developing a CBC was 2.01 %. As expected, the benefit of CPM decreased with age and stage. The maximum absolute benefit for a 50, 60, or 70-year-old woman with stage I breast cancer was 0.44, 0.25, or 0.10 QALYs, respectively. The respective maximum absolute benefit at age 40, 50, 60, or 70 for a woman with stage II primary breast cancer was 0.40, 0.28, 0.16, or 0.05 QALYs, and for a woman with a stage III primary breast cancer was 0.14, 0.08, 0.03, or –0.03.

With all utilities set to 1 (i.e., without incorporating quality of life in the analysis), the maximum absolute difference in life expectancy was 0.77 life years when the CPM benefit was 100 % and the annual probability of developing a CBC was 1.68 % in women with a first-degree relative with bilateral breast cancer. The results were not sensitive to the first-year utility for CPM. For example, a threshold analysis of the first-year utility for CPM for a 40-year-old woman with stage I breast cancer and no family history of breast cancer indicated that a first-year utility above 0.482 (compared with 0.90 for a unilateral mastectomy) would always result in CPM being the optimal preventative strategy. In the probabilistic sensitivity analysis for 40-year-old women with stage I breast cancer and no family history of breast cancer, 100 % of the trials had more effective CPM than no CPM irrespective of family history.

## Discussion

CPM is performed most frequently among women with a family history of breast cancer, *BRCA1/2* mutation status, and younger age at diagnosis [34–36]. The greatest gain in quality-adjusted life expectancy was among women age 40 with an ER-negative, stage I breast cancer and a first-degree relative with either a unilateral or bilateral breast cancer. However, our modeling results showed that for women age 50–60 with a stage I, II, or III breast cancer, CPM had a minimal benefit on quality-adjusted life expectancy among those with a unilateral, first-degree or second-degree family history of breast cancer. CPM had an unfavorable quality-adjusted life expectancy for most subgroups of women age 70 with a stage II–III, ER-positive or ER-negative breast cancer, regardless of the degree of family history of breast cancer. Familial history is thought to account for 15–20 % of all breast cancers [5], suggesting that CPM will benefit only a modest number of patients.

The determination of *BRCA1/2* mutation status among women with a personal history of breast cancer and a strong family history of breast cancer has increased in importance [37]. Life expectancy gains with CPM among women with a highly penetrant BRCA mutation diagnosed with breast cancer at age 50 has been estimated at 0.9 and 0.7 years for node-negative and node-positive disease, respectively [11]. In our model, women age 40 with a stage I (range 0.38–0.82) or stage II (range 0.28–0.65) breast cancer with a first-degree family history of breast cancer showed similar gains in life expectancy. Lester-Coll et al. [10] conducted a decision analysis to evaluate women age 45 by tumor subtype and cancer stages I–III, and suggested that CPM would not improve quality-adjusted life expectancy for the majority of women; however, family history was not included in their model. A biological explanation for our finding may be forthcoming because there is a growing trend towards expanding genetic testing in women with strong family histories and early-onset breast cancer who are not *BRCA1/2* mutation carriers to identify other germline breast cancer susceptibility mutations, such as p53, PTEN, PALB2, and CDH1 [38–40].

Although having a strong family history increases the risk of CBC, a recent study comparing outcomes among women age 41 or younger with breast cancer showed that having a family history of breast cancer did not worsen overall survival [41]. Arrington et al. [20] also argued that the survival curve for long-term survivors approaches that of the general population within 10 years. We showed that the maximum 20-year absolute overall survival rate benefit of CPM was 1.21 % for women with a family history of a first-degree relative with bilateral breast cancer. These results are consistent with prior decision models that have not considered family history and have shown a less than 1 % 20-year overall survival benefit for CPM [7]. Despite the minimal overall 20-year survival rate benefit of CPM, we found that the 20-year absolute DFS rate benefit ranged from 0.93 % in women with no family history to 12.59 % in women with a first-degree relative with a bilateral breast cancer. Given the difference in magnitude for the absolute 20-year overall and disease-free survival rate benefit for CPM, additional information such as quality-adjusted life expectancy should be considered in the decision-making process and may ultimately play a significant role in a woman’s decision of whether to have CPM.

Our decision model applied individual-level simulation to compare quality-adjusted health outcomes of CPM and surveillance only (no CPM). A strength of this analysis method was that it enabled us to estimate individual patient outcomes, such as the development of a CBC. As with all modeling studies, ours was subject to several limitations. Although our model considered a 10-year time frame in which patients were at risk for dying from the primary cancer, in reality breast cancer patients remain at risk of dying from their breast cancer after this time period, although the risk is reduced drastically [30, 42, 43]. In our model we relied on probability estimates obtained from the literature, particularly those concerning the risk of CBC and utilities for health states. Since the increased risk of CBC associated with ER-negative cancers as it relates to family history is not explicitly stated in the literature, our estimate of this risk factor was based on a calibration of plausible CBC risk ranges from reported data. Nevertheless, as expected, sensitivity analysis showed an increase in the benefit of CPM as the risk of CBC increases. Although health state utilities are dependent on the method used to obtain them, a consistent theme has emerged in the literature for prophylactic mastectomy. Studies have shown that women report negative feelings regarding body image [44] and adverse psychosocial outcomes following CPM which may not be accounted for in utilities available in the literature [45, 46], which we relied on. However, beyond the initial year after surgery, utilities for CPM are not significantly different from utilities for primary mastectomy [47]. In addition, a recent study showed that perceptions of CBC risk attenuated over time for both CPM and non-CPM patients [48].

## Conclusion

CPM reduces the risk of CBC and is associated with a gain in quality-adjusted life expectancy and disease-free survival in younger women who have an early-stage breast cancer and a history of breast cancer in first-degree relatives. However, even in this subgroup, CPM offers only a minimal overall survival advantage and the long-term psychosocial outcomes are unclear. Patients with ER-negative breast cancer consistently have a higher gain in quality-adjusted life expectancy than patients with ER-positive cancer, and this highlights the importance of anti-estrogen therapy as an alternative strategy for CPM among women with ER-positive breast cancer. Because patients are concerned about their lifetime experience of cancer, specifically driven by fear of developing a second cancer in the unaffected breast [49], quality of life and psychosocial considerations should be considered along with these clinical factors in surgical decision-making regarding CPM.

## Abbreviations

*BRCA1/2*: Breast cancer 1 or 2 gene
CBC: Contralateral breast cancer
CDH1: Cadherin-1 gene
CPM: Contralateral prophylactic mastectomy
ER: Estrogen receptor
p53: Tumor suppressor protein
PALB2: Partner and localizer of BRCA2
PTEN: Phosphatase and tensin homolog
QALY: Quality-adjusted life year
